# From *Psychologische Forschung* to *psychological research*: a rough journey through a century

**DOI:** 10.1007/s00426-021-01573-8

**Published:** 2021-08-06

**Authors:** Herbert Heuer

**Affiliations:** Leibniz Research Centre for Working Environment and Human Factors, Dortmund, Germany

## Abstract

*Psychologische Forschung* started as a journal “für Psychologie und ihre Grenzgebiete” and became strongly associated with the Berlin school of Gestalt psychology. Parallel to the fate of that school, the Journal was discontinued after 1938 and re-appeared only 1949. A number of years with variable and broad editorial boards and without a clear profile followed. In 1974 the Journal switched to English as the first German psychology journal and became *Psychological Research*. Gradually and without any abrupt changes—as indicated e.g. by analyses of citing and cited journals—the current profile as “An International Journal of Perception, Attention, Memory, and Action” was developed. During the last one to two decades the number of papers, the number of contributing countries, and the impact increase.

## Introduction

Why should one write a paper on the history of a journal? When I pondered this question, two reasons came to my mind. The first one is a somewhat personal one, the person-journal relationship. In his first editorial, the current Editor-in-Chief wrote: “this is not just any journal, but my first love—journal-wise” (Hommel, 2009). There may be several psychologists who share this (or an emotionally equivalent) person-journal relationship, and one of them is me. The relationship is based on papers spanning a time period of 40 years and on editorial-board service for more than 20 years. The second reason is less personal: *Psychological Research* has a history which reflects not only the evolution of psychology throughout the century, but also the political upheavals in Germany. In short: the Journal is a survivor of both political and scientific darkness.

History cannot be told objectively. It is always written from a certain perspective or even with a certain purpose, and occasionally it is re-written when the perspective or the purpose changes. This holds for the history of geographical regions, and on a smaller scale it also holds for the history of a science such as psychology or even of a journal such as *Psychological Research*. So this is a biased (or opinionated) report. And it is not the first one of the history of the Journal. The first 50 volumes, with volume 49 as the last one included in the data reported, have been reviewed by Scheerer (1988). Buchge (1994) provided a list of the editors of the Journal until 1992 (there are some deviations from the listings on the cover). The early history of the Journal—together with the history of the Berlin Psychological Institute—has also been sketched by Ash (1985), and its fate during the Nazi regime by Wohlwill (1987). Therefore, I shall deal with the history of the first 50 volumes (or 67 years) only briefly—but this against the counterforce of the storms that shook the Journal during those years, whereas more recently it floats on rather calm waters.

Fitts (1964) described three phases of skill acquisition—a cognitive, an associative, and an automatic phase. These phases became quite popular since then, which has always impressed me given their rather weak empirical foundation, namely the observation of learners and interviews with instructors (cf. Fitts & Posner, 1967, p.11). The popularity of this phasing seems to be based more on the intuitive sense it makes than on its empirical foundation. This example made me think about the phases in the history of *Psychological Research*. Unfortunately, this resulted in five rather than three phases. Although they are based on my observations only, I hope that they make intuitive sense for others as well. They will be designated as “awakening”, “destruction”, “comeback”, “consolidation”, and “rise”. For some of the boundaries between phases a year can be named, but other transitions between phases were more gradual. Similar to the identity of the person who acquires a skill, the identity of the publisher of the Journal remained the same throughout the phases of the Journal’s history—of course not as a person, but as a company.

## Awakening

The history of *Psychologische Forschung* begins on May 15, 1921, with a contract between the Springer-Verlag in Berlin and the initial group of editors, Kurt Koffka, Max Wertheimer, Wolfgang Köhler, Kurt Goldstein, and Hans Gruhle. This event had been preceded by negotiations and was followed by the first volume of the Journal, dated January 1st, 1922. The first article in this volume is “Zur Psychologie des Schimpansen” by Wolfgang Köhler, the second one “Untersuchungen zur Lehre von der Gestalt” by Max Wertheimer (which was followed by part II in volume 4, 1923). These two papers are in line with the popular belief that *Psychologische Forschung* was the journal of Gestalt psychology, specifically of the Berlin school. But the third paper, “Tod und Leben bei den Kpelle in Liberia” by Diedrich Westermann, makes it clear that the Journal was not intended as an in-house journal. In fact, the subtitle of *Psychologische Forschung* was “Zeitschrift für Psychologie und ihre Grenzgebiete” (“Journal of Psychology and its Associated Disciplines”), and the scope—as outlined on p.1 of the first issue—emphasized that “papers will be accepted on the basis of achievement rather than school affiliation” (cf. Scheerer, 1988). Among the associated disciplines, psychopathology was emphasized; the editors Gruhle and Goldstein represented the fields of psychiatry and neurology/neuropsychology. This emphasis is possibly related to the important role of medical publications for the publisher at the time.

Despite its broadly defined scope, the image of the early *Psychologische Forschung* as the journal of the Berlin school of Gestalt psychology is somewhat justified for mainly two reasons. First, in the early twentieth century in-house journals were not uncommon in Germany. As described by Ash (1985), after World War I the Berlin institute became well-equipped with rooms and financial support. Next to the Leipzig institute, founded by Wilhelm Wundt and headed by Felix Krueger since 1917, the Berlin institute became another German institute of high visibility that attracted students from abroad. The Leipzig institute had its own journals over the years (*Philosophische Studien*, *Psychologische Studien*, *Neue Psychologische Studien*—in this order), so this fact may have strengthened the tendency to associate the Berlin-based journal with Gestalt psychology.

Second, according to Ash (1985), over the years the image of *Psychologische Forschung* as the journal of Gestalt psychology became more and more justified by the published papers. This is evident from a list of pre-war papers that still received citations between 1971 and 1980 (Scheerer, 1988). Most of these ‘surviving’ papers, often with Berlin-based authors, have the theoretical background of Gestalt psychology. This is a selection of classic contributions: Zeigarnik (1927) and Ovsiankina (1928) on consequences of interrupted actions, Duncker (1929) on induced motion, von Restorff (1933) on memory for isolated items, Karsten (1928) on “psychische Sättigung” (a notion that is probably more popular in German than in English), and further papers by Wertheimer (1923), Gottschaldt (1926), Metzger (1935), Lewin (1926)—authors and phenomena strongly associated with Gestalt psychology (though Lewin is not that prototypical).

In the second half of the twentieth century, internationalization became a prominent issue both for the Journal and for German psychology in general (cf. Gigerenzer et al., 1999). In this respect it is noteworthy that *Psychologische Forschung* published a number of papers in English—in spite of the German-speaking editorial board, the German publisher, and the strong influence of the Berlin institute. According to Scheerer (1988), these were 38 papers in the pre-war period (about 15% of all papers).

## Destruction

The Berlin group of Gestalt psychologists was dissolved after 1933, mainly by emigration. Köhler’s efforts to defend the institute against the Nazi administration are rather well documented (see Ash, 1985, and other sources listed there), but in the end they were not successful. Köhler himself emigrated in 1935. The fate of the Journal was almost parallel to that of the Institute, except that it ceased to exist rather than being brought into line politically. The group of editors of the Journal had been expanded by Gelb in 1930; in 1933 Goldstein was no longer listed as a member of the editorial board, and for volumes 21 and 22 (1937/38) only Köhler remained, already as an emigrant. Subsequently, he insisted on the suspension of the Journal, for some time at least. This period of silence distinguishes *Psychologische Forschung* from other German psychology journals of the time.

According to Wohlwill (1987), the fate of German psychology journals during the Nazi regime was pretty much shaped by the editors. Köhler had requested that the journal should be discontinued because no more papers based on work that had started in Berlin could be expected, and because he could not urge his students in the USA to publish in a journal that would not reach the relevant US audience. In response to a letter of the publisher, who had asked Köhler to reconsider this decision, he wrote (in the translation of Wohlwill, 1987, p. 179):

“…This reason still remains in effect. I don’t doubt that it might be possible through all sorts of compromises to continue the journal in a fashion that is not in keeping with its past. But I do not wish to have any part in that, and I suspect that would be a disservice to the publisher, as much as to anyone else.

The situation has incidentally become even more grave in the meantime, as I have been notified that further contributions by Dr. Wallach would no longer be accepted. That is of course a decision that does not affect Dr. W. alone. I know, it is not your decision, Dr.; but that does not change the fact that no editor can permit interference from nonsubstantive influences in the selection of the content of a scientific journal. I did make certain concessions while there was still a question of maintaining for our last students the only appropriate place for their publications. However, since their studies have appeared there no longer remains the slightest excuse for me to subject myself to offenses against the ethics of science.”

According to Scheerer (1988), the concessions to which Köhler refers are probably related to the changes in the editorial board. Köhler’s strictness regarding the Journal and its acceptance criteria contrasts with the policies pursued by other German psychology journals and their editors. As described by Wohlwill (1987), some of the editors adjusted to the Nazi ideology as far as it appeared necessary (or suitable) for survival. Others, however, became strong advocates of the ideology, even trying to fuse it with their own theorizing (with Jaensch, the editor of *Zeitschrift für Psychologie*, representing an extreme of the fusion of psychological theorizing with Nazi ideology; cf. Merz, 1960, p. 89).

*Psychologische Forschung* and the Berlin school of Gestalt psychology shared the same fate during the Nazi regime, and this contrasted with the fate of the Leipzig school of “Ganzheitspsychologie” (cf. Prinz, 1985). The difference between the schools is remarkable because of their commonalities, which included anti-elementarism and an emphasis on Gestalts. Ideas like that could easily be related to elements of the Nazi ideology such as the notion of a primacy of the nation (or race) over the individual. In fact, the Leipzip group was in favor of the Nazi ideology, at least during the early years and probably without sharing its antisemitism (Felix Krüger, the head of the Leipzig group, retired early in 1938, more or less pressed by the Nazi administration). Aftermaths of the political affinities of the Leipzig school became virulent again about 15 years after the end of the Nazi regime when prominent previous members were still influential in German psychology (cf. Merz, 1960, 1961; Wellek, 1960). One reason for the different fates of the Berlin and the Leipzig schools is obvious: major scientists of the Berlin group were Jewish, those of the Leipzig group were not. But this does not explain the difference in intellectual distance to the Nazi ideology. This is likely related to differences in the style of theorizing, only imperfectly characterized by “irrational vs rational”. These differences become pretty obvious simply from reading papers by Krüger (e.g. 1926) and by Köhler (e.g. 1969). Obviously, there was a kind of psychological theorizing in the first half of the twentieth century in Germany that was susceptible to ideological deformations—but that was not the theorizing represented by *Psychologische Forschung*.

## Comeback

According to Scheerer (1988), the initiative for the comeback of *Psychologische Forschung* had been taken by Johannes von Allesch and Hans Gruhle. Gruhle was a member of the pre-war editorial board, but the other surviving former editors, Köhler and Goldstein, apparently had not been contacted. Von Allesch had been an assistant at the Berlin institute both before and after World War I and had published in the Journal, but was not a typical Gestalt psychologist. At the time being he was professor in Göttingen (retired in 1948), and he served on the editorial board for only one volume. In addition to the psychiatrist Gruhle, who also served for the next volume, the first post-war editorial board consisted of five psychologists (von Allesch, Düker, Heiss, Lersch, and Metzger), and an anthropologist (Thurnwald). Among the psychologists, both Heiss and Lersch had a non-empirical orientation and published primarily on “Charakterologie”, a kind of personality research. Only Düker and Metzger were experimental psychologists. Metzger was one of the few non-emigrated Gestalt psychologists (cf. Stadler, 1985) and one of the first post-war professors at Münster University in 1946. There he re-built the psychological institute, beginning with 16 m^2^, by “Eroberungszüge in vorhandene Gebäude” (“campaigns of conquest into existing buildings”) (Metzger, 1972, p. 203). Düker had been a student of Narziß Ach in Göttingen and had survived Nazi prosecution (including the concentration camp Sachsenhausen); he established scientific Psychology in Marburg 1946 (after the Jaensch professorship and a few interim years during the war) and solved the housing problem by renovating barracks left by the British military government (Düker, 1972, p. 61), where the institute still is (after a couple of additional renovations). In the hunger years after the war Düker established the “Mettessen” (a meal of minced pork with bread) of the institute’s staff, which became an annual event for decades. (I am aware that this has almost nothing to do with the history of *Psychologische Forschung*, but with the person and the time of the Journal’s comeback.)

The first post-war issue (volume 23/1–2) appeared on January 1st, 1949, with five papers; the following three issues of volume 23 with ten papers in total appeared 1950 and 1951. Of the first five papers, three were of an experimental nature, two with a Gestalt background by Rausch and Cymbalistyj, a student of von Allesch. The third one was by Düker. The final two papers were nonexperimental, written by Gruhle and by Thurnwald. Thus, there was a mixture of topics and theoretical as well as methodological approaches. As noted by Scheerer (1988) and Engelkamp (1996), there was no clear profile of the Journal in the post-war period. Just as another example of the variety of published papers, consider volume 26/3 of 1961 with four articles. The first one is by Schmidtke: in the article (“Zur Frage der informationstheoretischen Analyse von Wahlreaktionsexperimenten”) he refers to the Hick-Hyman law and, in addition to findings on practice and fatigue, shows that it breaks down with too many choice alternatives. The second paper is by Traxel and Heide (“Dimensionen der Gefühle”) with an empirical part based on similarity judgments and factor analysis. These two papers can be seen as representatives of Cronbach’s (1957) “two disciplines of scientific psychology”, experimental and correlational psychology. The third paper by Otfried Spreen (“Konstruktion einer Skala zur Messung der manifesten Angst in experimentellen Untersuchungen”) was of an applied nature and concerned with the development of a diagnostic scale. The fourth paper was on “Further studies on the stellar orientation of nocturnally migrating birds” by Sauer, a topic quite remote from those of the other papers, but fitting to the subtitle of the Journal which from 1959 to 1967 (Vol. 26–30) was “Journal of Experimental Psychology, Ethology, and Medical Psychology”. With its broad multidisciplinary orientation and its variegated (or absent) profile, the Journal stabilized to some extent and again attracted English papers. According to Scheerer (1988), these were 12.8% in the period between 1949 and 1965 (Vol. 23–29) and 53.3% in the period between 1966 and 1974 (Vol. 30–36).

The year 1967 (vol. 31) saw major changes of the editorial board. Two of the new editors were from the USA: Richard Held of MIT, who had spent some time with Köhler at Swarthmore College, and Herschel Leibowitz, also a prominent researcher in the field of visual perception. Three of the new editors from Germany were post-war psychologists: Theo Herrmann, who moved to Marburg in 1968, Hans Hörmann, who moved to Bochum in 1969, and Karl-Hermann Wewetzer from Giessen. Neighboring disciplines were represented by Otto-Joachim Grüsser, a neurophysiologist from Berlin, and three ethologists, Paul Leyhausen, Detlev Ploog, and Franz Sauer. There was also a new subtitle of the Journal in two languages: “Empirische Untersuchungen von Grundproblemen der Psychologie, Basic Research in Psychology”, which seems to imply the inclusion of findings from neurophysiology and ethology.

The year 1967 is memorable for two more reasons. First, it is the year that Wolfgang Köhler died. The obituary in volume 31/1 had contributions by Rudolf Bergius, who represented the Deutsche Gesellschaft für Psychologie, Hans Hörmann, and Hans-Lukas Teuber, then head of the psychology department at MIT (and born in Berlin). Teuber reports a statement by Köhler that I repeat here because it has some relevance for a debate that came a few years later: “You know, Lukas, it’s really amazing, how many unclear things you cannot say in English.” The obituary is followed by Köhler’s last paper, the manuscript for an invited address to the APA. Second, in the same volume (31/1 and 31/4) four papers appeared which grew out of oral presentations at a symposium held in Boston, Mass. They are by Schneider, Ingle, Trevarthen, and Held and espouse the notion of two visual mechanisms (or two visual systems), one for WHAT we see and one for WHERE we see it. This notion became quite popular (and modified) later on (e.g. Milner & Goodale, 2008).

In the next decade or so the board of editors grew larger and became more international. This large editorial board served to mark the scope or profile of the journal and its reputation (the impact factor did not yet serve that function), also to attract authors on certain topics and from certain regions. These are outward-directed functions of an editorial board. But there is also routine work to be done. According to Scheerer (1988), these editorial duties had mainly been assigned to Hans Hörmann and Klaus Foppa in the years after 1967, without this being marked in any way in the listings of the editorial board. Probably at some time in the early 1970-ies the peer-review system was introduced. From Scheerer (1988) we know it was there in 1979, and as an author he had experienced it already in 1973.

The next major step in the comeback of the Journal was vol. 37 (1974/1975). Robert B. Freeman was appointed as what was now called “Coordinating Editor”, and in vol. 39 he was listed as such. He had moved from Penn State to Konstanz University in 1972. In an unsigned editorial in vol. 37/1 it is said: “To accentuate the international character of *Psychologische Forschung*, we not only decided to change the subtitle to reflect the emphasis found in editorial material, but also to translate its original title, so that henceforth the journal will be known as: *Psychological Research*, An international Journal of Perception, Learning and Communication, Founded as *Psychologische Forschung*. For the same reason we invited several distinguished colleagues from abroad to join the Editorial Board.” It is emphasized that the change in title does not imply a change in publication policy, and that neuropsychological, psychophysiological, and ethological contributions are welcome. “It is this very open-mindedness that seems to us so necessary for the advancement of psychology as a science.” Whereas vol. 37 still had four papers in German and vol. 38 one, from vol. 39 on all papers were in English.

As reviewed by Krampen et al. (2012), *Psychologische Forschung* was the groundbreaker among German psychology journals in the process of anglicization. Other journals followed with a delay, most of them only after the turn of the millennium. The change of language stirred a lively debate, briefly reviewed by Scheerer (1988). Among the arguments were that being forced to publish in English might prevent the expression of subtle details by native German speakers and bring them into an inferior scientific position simply by virtue of language deficits. (In this regard the above cited statement of Köhler on the difficulty to express ideas in English that are not yet clear for oneself might be relevant.) On the other hand, it was emphasized that papers written in German would find no readership in English speaking countries. Whatever the validity of these arguments, the analysis of Krampen et al. (2012), that considered several characteristics of papers written in the 4-year periods before and after the change of language as well as their impact in the subsequent 2 years, revealed almost no differences for *Psychologische Forschung/Psychological Research*; only the language of citing articles was more often English after the change than before. This essentially absent short-term effect of switching from German to English, however, cannot be generalized across other journals.

From volume 41 (1979) on, Eckart Scheerer served as the Coordinating Editor which he had actually started in 1978. For him the person-journal relationship was a quite close one (cf. Heuer & Prinz, 1997). First, it was related to the history of the Journal. As an indication, Scheerer (1991) did not only remark that the change to English might have been too early because German-speaking authors were not yet prepared to publish in English, but also that the new name *Psychological Research* might hide its history as *Psychologische Forschung*. Second, the broad scope of the Journal matched the broad interests of Eckart Scheerer. Finally, his person-journal relationship might also have been nourished by early papers of him such as the one with Bischof in 1970 that on slightly more than 80 pages presented an analysis of the perception of the vertical based on visual and vestibular input. Although Scheerer started his editorship with a large editorial board (Broadbent, Engelkamp, Flores d ‘Arcais, Gross, Held, Hurwitz, Johansson, Leibowitz, Oleron, Tulving), the bulk of the work—with the exception of occasional support—rested on him. Only about 10 years later, in 1988, there was a major change in the editorial board that was followed by a more distributed processing of manuscripts: only Broadbent and Engelkamp remained on the board, and Bridgeman, van der Heijden, Heuer, and Mewhort came in.

When Eckart Scheerer ended his period as Coordinating Editor in 1991, but continued to stay on the board, he wrote: “After about 12 years of editorial work on the journal, I would like to see something meaningful in my activities as an editor. (History is the attempt to render meaningful what is meaningless.) Is there anything that has been accomplished during these years? Future historians of science will have to decide. In my own view, the most important thing is that the journal was kept alive.” (Scheerer, 1991). But he also noted that the journal was not only kept alive, but has had increasing numbers of citations during the last couple of years. Thus, in terms of the phases of the Journal’s history, at some time during the editorship of Eckart Scheerer the comeback phase had turned into the consolidation phase.

## Consolidation

Following Eckart Scheerer as Coordinating Editor, I had the opportunity to continue the consolidation phase. From vol. 53 (1991) on, the compound name *Psychological Research/Psychologische Forschung* (on the cover until 2006, on first pages of articles until 1993) was used to make it clear that *Psychological Research* is the same Journal as *Psychologische Forschung*. The success of this measure appears somewhat mixed. For example, in the Journal Citation Reports (Clarivate Analytics) the compound name of the Journal appears, whereas for a Google Scholar search with Harzings’s Publish or Perish only the German and English names used separately produced meaningful results. Beginning in 1991, there was also a modification of the subtitle: “An International Journal of Perception, Cognition, and Action”. After Johannes Engelkamp resumed the Coordinating Editorship in 1996, the subtitle was made more specific in 1998 (vol. 61): “An International Journal of Perception, Attention, Memory, and Action”. This covered quite well the topics of the papers in the Journal (Engelkamp, 1996).

There is one aspect of the publication strategy that was initiated by Eckart Scheerer in 1980, continued throughout the consolidation phase, and is still present. This is a series of Thematic Issues, with manuscripts being subjected to a normal review process. A kind of precursor, though published across separate issues of a volume, are the four papers on two visual systems mentioned above. Thematic Issues often, but not always, grew out of conferences and were mainly handled by guest editors. On average, there was almost one Thematic Issue per volume since vol. 51, with the majority of them on topics in the general field of perception and action.

Thematic Issues serve three functions at least. In the comeback and consolidation phases, there could be problems with too few submissions. Thematic Issues would help, but it could happen that more submissions arrived than expected, leading to an extra volume at least once (cf. Heuer, 1995). The second function of the Thematic Issues was and is to contribute to the profile of the Journal, which again was particularly important in the comeback and consolidation phases when the scope became progressively focused. Therefore the editors sometimes adopted an active role in inviting manuscripts for Thematic Issues they thought appropriate. Examples are “The Simon effect” (vol. 56/1, edited by Carlo Umiltá) or “Executive processing” (vol. 63/3–4, edited by Gordon Logan). Finally, Thematic Issues were a means to invite contributions by well-known authors and thereby increasing the reputation of the Journal. (At the times of a huge reputation gap between German-based Journals such as *Psychological Research* and APA journals such as the *Journal of Experimental Psychology* series, this strategy for receiving excellent submissions may have failed occasionally as a manuscript finally submitted upon invitation could be of a kind the author thought appropriate for a low-level journal; I have to admit that my memory here may actually refer not to real events, but only to risks discussed at one or the other occasion.)

**Fig. 1:**
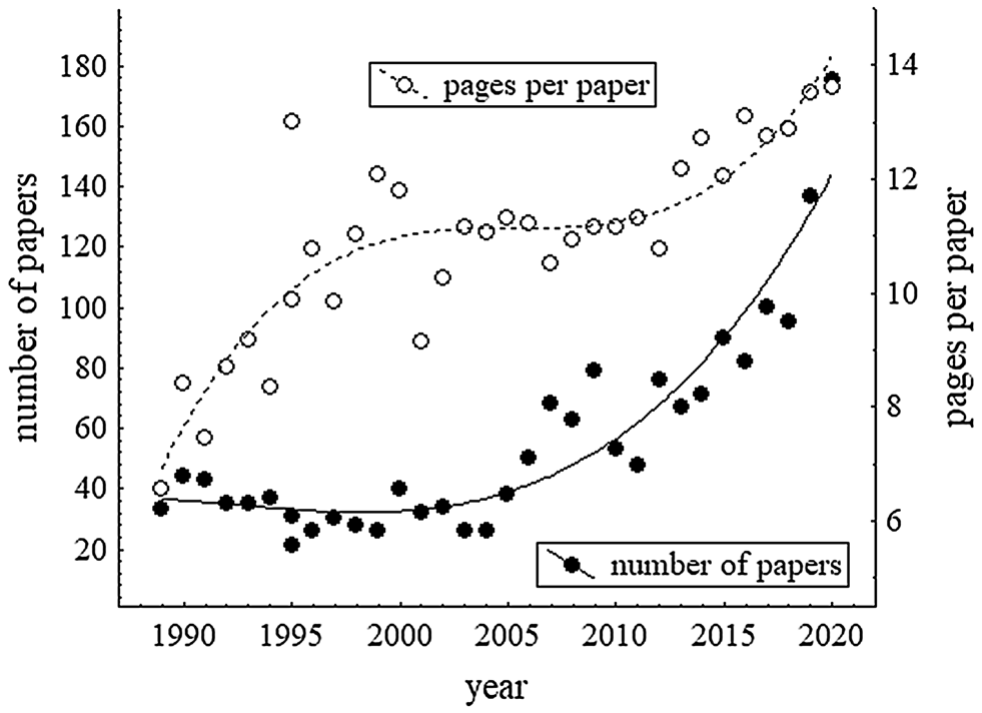
Number of papers and number of pages per paper for the years 1989–2020. These are numbers of papers per volume; although volumes do not always correspond to years exactly, they do roughly so; only for the year 1995 there was an additional volume. The continuous and broken lines are third-order polynomials fitted to the data points

Problems with too few submissions to fill a volume, as they were present during the comeback phase and still at the horizon during the consolidation phase, disappeared towards the end of the last millennium. In Fig. 1 the development of the number of papers per volume is shown. In the 17 years between 1989 and 2005 there were about 33 papers per volume—there were actually 18 volumes in this time period. Thereafter the number of papers per volume increased—which leads to the next phase, the rise.

Before turning to that phase, however, I want to point to the increasing number of pages per paper, which is evident from Fig. 1. The number of pages increased until about 2000. In 2001 both Engelkamp (as outgoing Coordinating Editor) and Frensch (as incoming Coordinating Editor) (2001) expressed their concern about this tendency (which, as noted by Frensch, was not restricted to *Psychological Research*). The trend stopped for the next dozen years or so, but the target length of 8 pages that Engelkamp (2001) mentioned was never reached on average. During the last about 10 years the length of papers continued to increase. As pointed out to me by Bernhard Hommel, this might be due—at least in part—to increasing requests of reviewers to specify procedural details, to give justifications of specific analyses etc. In this respect a comparison of the word counts of original submissions and finally accepted manuscripts might be illuminative (for this manuscript the counts were 8771 and 8993, respectively). Whatever the reasons for the increasing length are, it might not be desirable. Researchers are faced with a growing number of relevant papers, and undue lengths might be deterrent. A number of journals have length limits, and several high-impact journals have short articles together with elaborate illustrations. The situation is somewhat reminiscent of a famous statement that comes in different varieties and is attributed to different authors (my original memory is Kleist, but this may not even be the most popular reference): it roughly says “I am writing a long letter because I do not have the time for a short one”. It might also be worth reminding of the final sentence of the first editorial of *Psychologische Forschung* in 1922 (translation by Scheerer, 1988): “The times require that the writing be as tight and the reasoning as stringent as possible.”

## Rise

The last phase of the Journal, which leads into the present days, begins in the early years of the millennium and is marked by an increasing number of papers. A couple of more technical changes brought the Journal in line with current standards. In 2001 “Online First” was introduced, the rapid electronic and citable publication of accepted papers before they are assigned to a volume. The option to publish Open Access was introduced in 2008, and an electronic submission system was introduced in 2009 when Bernhard Hommel succeeded Peter Frensch as editor. At that time the “Coordinating Editor” became “Editor-in-Chief”, and the other members of the editorial board became “Associate Editors”, consistent with common denominations.

**Fig. 2:**
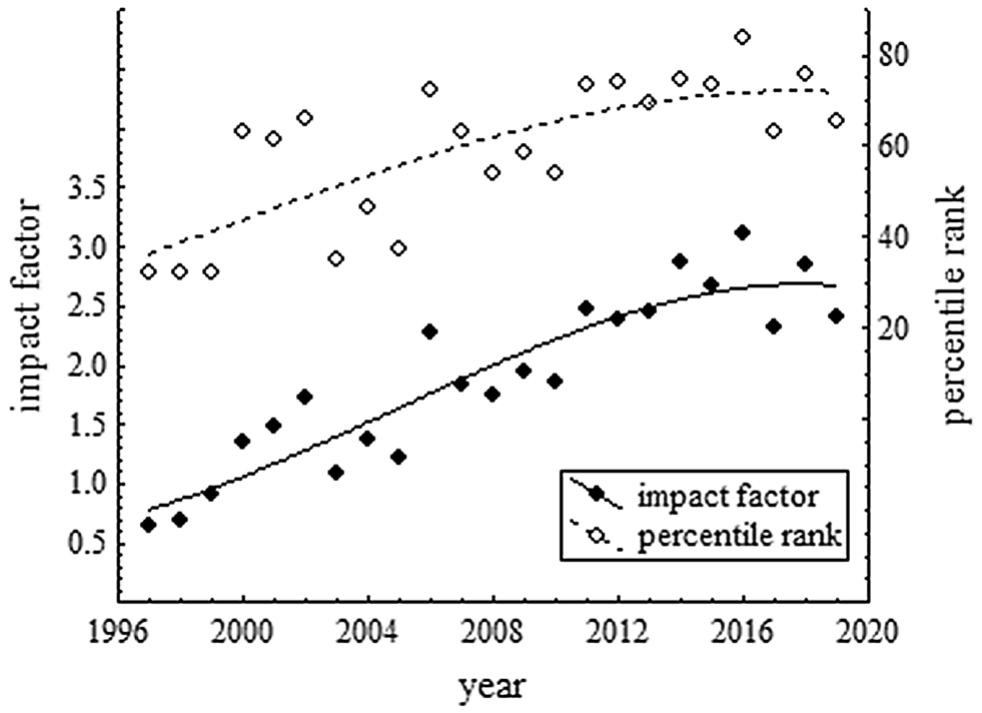
Time course of the impact factor and the percentile rank of *Psychological Research* among the 89 journals of the category PSYCHOLOGY, EXPERIMENTAL (source: Clarivate Analytics, Journal Citation Reports). The continuous and broken lines are third-order polynomials fitted to the data points

The current phase of the Journal is not only distinguished by the increasing number of published papers, but also by its increasing impact factor as illustrated in Fig. 2. In principle the increase could reflect a general increase of impact factors. However, it is not only the impact factor that increased during the last 20 years, but also the Journal’s percentile rank among the 89 journals of the category PSYCHOLOGY, EXPERIMENTAL of the Journal Citation Reports (the top journals in this category are *Trends in Cognitive Sciences* and *Nature Human Behaviour*). At least in terms of impact factors, the huge reputation gap of the past between *Psychological Research* and APA Journals such as *Journal of Experimental Psychology: Human Perception and Performance* has been reduced considerably (and in some years even be reversed).

*Psychological Research* has always been an international journal, even when it was *Psychologische Forschung* and most papers were in German. Nevertheless, the decision to publish in English only had also been motivated by the objective to boost contributions from other than German-speaking countries. Therefore both Scheerer (1988) and Engelkamp (1996) reported relevant data that are repeated in Table 1 together with recent data taken from the Journal Citation Reports. From Table 1 some trends appear fairly robust. First, the number of countries with contributions to the Journal is steadily increasing. Second, for some countries there is a steady increase of the percentage of contributions: Italy, France, Belgium, Spain (and “other countries”). Third, for some countries there is a steady decline of the percentage of contributions: Germany and Sweden. For the USA there is a strong decline in the last time period, and for the Netherlands a less conspicuous decline; the reliability of these and other non-monotonic changes may be more questionable than the reliability of the monotonic ones.

**Table 1:** Percentages of contributions to *Psychological Research* from different countries

Country	Vol. 81–83	Vol. 48–57	Vol. 40–49
Germany	19.30	24.85	30.43
United Kingdom	12.32	8.08	14.05
USA	10.88	20.06	18.73
Canada	8.21	8.08	8.36
Italy	7.80	3.29	2.01
Netherlands	6.78	11.38	8.36
France	5.13	3.29	1.00
Israel	4.72	1.20	2.34
Belgium	3.90	2.99	1.34
Switzerland	3.49	0.60	1.34
Spain	2.67	0.60	0
Australia	2.26	5.69	1.34
China	1.85	0	0
Japan	1.64	0.90	3.68
Sweden	1.04	2.99	3.01
Other countries	8.03	6.00	4.01
# of contributions	487	334	299
# of countries	36	21	18

For volumes 40–49 and volumes 48–57 the data are from Scheerer (1988) and Engelkamp (1996), for volumes 81–83 the data are from Journal Citation Reports. Countries are ranked by the most recent percentages for all countries with more than 1% of the contributions.

The increasing proportion of contributions from countries other than German-speaking ones is in line with the objectives of anglicization of the Journal. Beyond this, it is tempting to speculate about the reasons for the more specific developments. Countries with increasing percentages of contributions are primarily countries in which Romance languages prevail, such as Italy, France, Spain, and parts of Belgium; whereas, countries with declining percentage contributions tend to be countries in which Germanic languages prevail, such as Germany, USA, and the Netherlands. Given the increasing number of published papers across the years, declining percentages might be just a consequence of increasing numbers of contributions from countries with increasing percentages. Possibly the difference between the two language families reflects their distance to English and—related to this—differences in the time course of accepting English as the lingua franca of scientific communication in psychology.

Similar to groups of scientists, groups of journals have a structure which is marked by mutual referencing. Scheerer (1988) started to inquire into the family of Journals to which *Psychological Research* belongs. In Table 2 those journals are listed that were cited in the years 1986/87 (volumes 48 and 49) according to Scheerer together with those Journals that were cited in 2019 according to the Journal Citation Reports. In setting up this table, I have taken name changes of journals into account. For example, *Attention, Perception, & Psychophysics* is still more often cited under its previous name *Perception & Psychophysics* than under its current name, and citations of the *Journal of Experimental Psychology* before it was split up in 1975 are added to the citations of *Journal of Experimental Psychology: General*.

**Table 2:** Ranking of journals with more than 50 citations in *Psychological Research* in 2019 (source: Journal Citation Reports) and ranks of journals with less citations listed in Table 5 of Scheerer (1988). For comparison the ranks of the journals for the years 1986/87 are given (ranks among 30 journals, neglecting the conference series “Attention and Performance” in Table 5 of Scheerer, 1988); in that column journals which appeared only 1986 or later are marked by “–-”, and journals which did not appear in Scheerer’s list by “???”

	2019	1986/87
*Journal of Experimental Psychology: HPP*	1	4
*Psychological Research*	2	8
*Psychonomic Bulletin & Review*	3	–-
*Attention, Perception, & Psychophysics*	4	1
*Psychological Science*	5	–-
*Consciousness & Cognition*	6	–-
*Frontiers in Psychology*	7	–-
*Journal of Experimental Psychology: General*	8	6
*PLoS ONE*	9	–-
*Neuropsychologia*	10	20
*Cognition*	11	22
*Journal of Experimental Psychology: LMC*	12	11
*Psychological Bulletin*	13	13
*Trends in Cognitive Sciences*	14	–-
*Psychological Review*	15	3
*Quarterly Journal of Experimental Psychology*	16	7
*Memory & Cognition*	17	2
*Experimental Brain Research*	18	23
*Acta Psychologica*	19	10
*Journal of Neuroscience*	20	???
*Proceedings of the National Academy of Sciences*	21	???
*Frontiers in Human Neuroscience*	22	–-
*Journal Cognitive Neuroscience*	23	–-
*Science*	24	14
*Behavior Research Methods*	25	???
*Neuroimage*	26	–-
*Psychology & Aging*	27	–-
*Cognitive Psychology*	28	9
*Journal of Personality and Social Psychology*	29	28
*Nature*	30	???
*Vision Research*	31	21
*Journal of Neurophysiology*	34	27
*Journal of Experimental Child Psychology*	35	24
*Perception*	43	18
*Behavioral and Brain Sciences*	49	16
*Perceptual and Motor Skills*	61	19
*British Journal of Psychology*	64	17
*American Psychologist*	69	25
*Journal of Memory and Language*	74	5
*American Journal of Psychology*	111	15
*Zeitschrift für Psychologie*	155	30
*Psychologische Beiträge*	223	26
*Canadian Journal of Psychology*	280	12
*Psychological Monographs*	–-	29

(For 2019 citations of *Psychological Research* includes *Psychologische Forschung*, *Journal of Experimental Psychology: General* includes *Journal of Experimental Psychology*, *Quarterly Journal of Experimental Psychology* includes *Quarterly Journal of Experimental Psychology-A*, *Attention, Perception, & Psychophysics* includes *Perception & Psychophysics*; *Psychologische Beiträge* includes *Psychology Science Quarterly* which has no impact factor but is assigned a rank according to four citations and alphabetical order among all journals with four citations and an impact factor).

A few trends in Table 2 are apparent. First, among the journals that rank high in 2019 several ones did not yet exist in 1986. Among them are prominent journals of scientific societies such as the *Psychonomic Bulletin & Review* of the Psychonomic Society and *Psychological Science* of the Association for Psychological Science. Among the new journals are also broad open-access publications such as *PLoS ONE* (since 2006) and two journals of the *Frontiers* series (since 2010 and 2007). (However, *Scientific Reports*, another broad open-access journal, starting in 2011, ranks only 89 in 2019). Second, higher ranks in 2019 can be observed for Neuroscience journals such as *Neuropsychologia*, *Experimental Brain Research*, and *Journal of Neuroscience* (with the exception of the *Journal of Neurophysiology*); among the new journals is the *Journal of Cognitive Neuroscience*. Third, journals dedicated to perception such as *Attention, Perception, & Psychophysics*, *Vision Research*, and *Perception* have lost ranks. Fourth, the German-language journals that also switched to English (*Zeitschrift für Psychologie* and *Psychologische Beiträge*) have lost considerably; the former *Zeitschrift für experimentelle und angewandte Psychologie*, later *Zeitschrift für experimentelle Psychologie* and now *Experimental Psychology* did not appear in the 1986/87 ranking, and has now rank 60.

The Spearman rank correlation between the rankings of the journals listed both for 1986/87 and 2019, which compresses the widely spread ranks listed in Table 2 for 2019 to the same range between 1 and 29 for both time periods, is 0.5. Thus, neglecting the journals that appeared only in the 2019 ranking, there is some similarity between the profiles of referenced journals across a time period of slightly more than 30 years. Differences between these profiles seem to reflect a declining weight of studies of basic perceptual processes among the papers published in *Psychological Research* and an increasing weight of neuroscience findings. The latter might reflect the increasingly tighter link between the traditional functional analyses of experimental psychology and the identification of the relevant neural correlates. In fact, strengthening of the cross-disciplinary links to the neurosciences is an explicit objective for the Journal (Hommel, 2009).

**Fig. 3:**
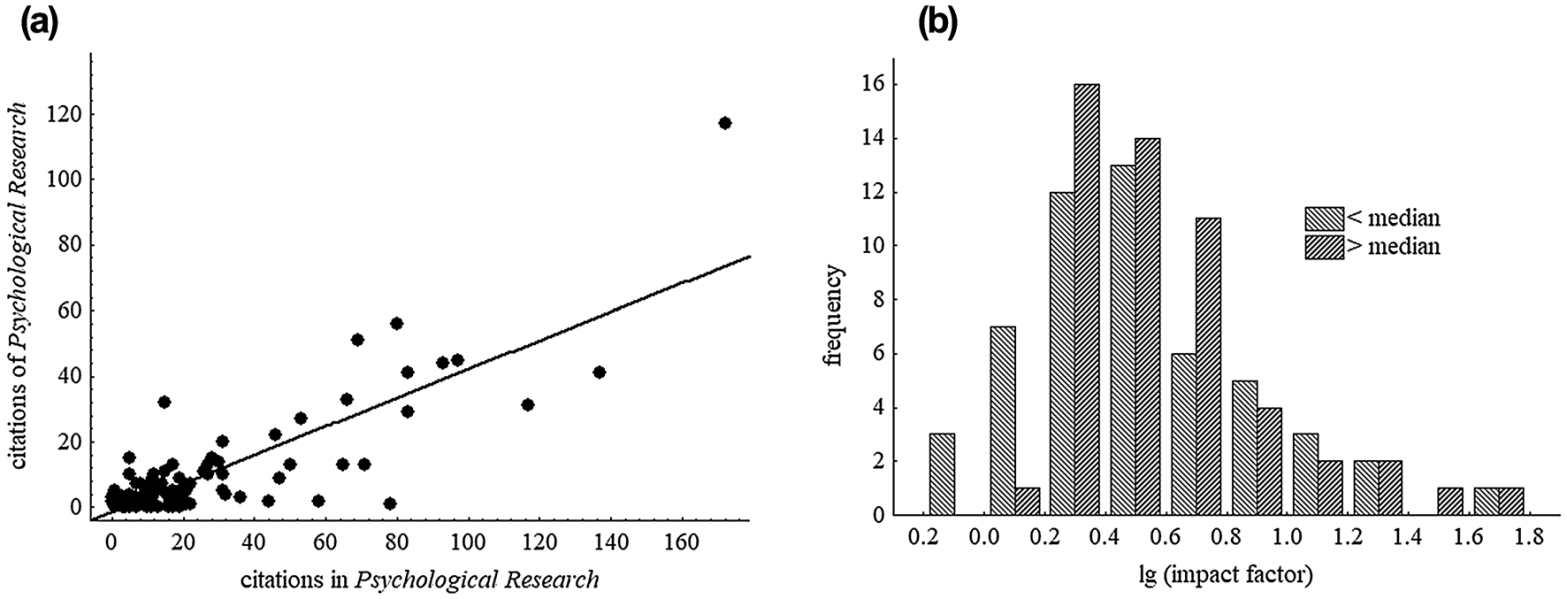
**a **Frequency of citations of *Psychological Research* in 108 journals as a function of the frequency of citations of these journals in *Psychological Research*. **b** Logarithmic impact factors of journal with differences between being cited and citing *Psychological Research* below and above the median of nine citations

What is the relation between the journals cited in *Psychological Research* and those that cite it? The following analyses are based on the citations of papers that appeared in the last 10 years, using the data provided by the Journal Citation Reports. First, I selected all 109 journals with 10 or more citations in *Psychological Research* in 2019. For these journals I also determined the frequencies with which they cited *Psychological Research*. Figure 3a shows the frequency of citing *Psychological Research* as a function of being cited for 108 journals (without *Psychological Research* itself, for which the frequencies of citing and being cited are trivially identical). There is a clear positive relationship, indicating a family of journals with similar mutual citation frequencies. The Spearman rank correlation is 0.61. Of course, this correlation is not perfect. Deviations may be chance, but there might also be systematic asymmetries. What distinguishes journals that are more often cited by *Psychological Research* than citing it from journals that cite *Psychological Research* more often than being cited by it?

Citing appears to be a somewhat delicate process that becomes even more delicate as the number of citable papers increases. When several papers could be cited in principle, a selection has to be made. Sometimes there are “standard references”, such as Fitts and Posner (1967) for the stages of skill acquisition mentioned above (according to Google Scholar this book is cited about three times as frequently as the original book chapter by Fitts, 1964). Otherwise there seem to be different practices, and at least some of them are somewhat off-the-record. One practice is the formation of citation pools—groups of labs or researchers with high mutual citation rates and neglect of other labs or researchers. Another practice is the definition of “non-existent literature”, which also helps in dealing with too many research papers. More recently, selection among multiple candidate references has also been hypothesized to be shaped by the first outputs of search systems such as Google Scholar. Finally, potential referees or editors might shape the selection of references (sometimes even the actual rather than the potential referees do it). In any case, the practices of referencing might easily result in asymmetries not only between persons, but also between journals. The impact factor of a journal should be a predictor of such asymmetries, for example because high-impact journals are less likely to be considered “non-existent” and more likely to be ranked high in search-system outputs. Even beyond the different practices of selecting references, one could expect higher-impact journals to be more frequently cited in lower-impact journals than the other way round, simply because of the difference in impact factors and thus citation frequencies.

Based on such considerations, I checked whether those journals that are more often being cited than citing *Psychological Research* are indeed those with a higher impact factor. Figure 3b shows the logarithms of the impact factors of journals above and below the median of the difference between the frequencies of being cited and citing *Psychological Research* (the median was 9; four journals with exactly this difference were omitted so that 52 journals remained for each group). Journals with an above median excess frequency of being cited in *Psychological Research* have a somewhat higher impact factor, indeed, but this difference is not impressive. (With the logarithms of the impact factors, which stretches the range of small and compresses the range of high impact factors, the difference between the distributions is actually better visible than with the impact factors themselves). A statistical comparison between the impact factors of the two groups of journals failed to approach statistical significance—thus the evidence for a citation asymmetry between journals with respect to the impact factor is weak at best. Given that papers in high-impact journals are cited more frequently than papers in low-impact journals, the absence of a clear citation asymmetry between journals may appear counter-intuitive. However, what applies to individual papers might not apply to journals, for example because the larger number of papers in lower- than in higher-impact journals can compensate for the difference in citation rates of individual papers.

In search of other factors that could possibly shape the citation asymmetry, I listed the journals with the strongest asymmetries. The six journals with the strongest asymmetry towards *Psychological Research* (more often being cited than citing *Psychological Research*) were all journals that did not yet exist in 1986, namely *PLoS ONE*, *Consciousness & Cognition*, *Psychological Science*, *Frontiers in Human Neuroscience*, *Trends in Cognitive Sciences*, and *Frontiers in Psychology*. However, another broad open-access journal, similar to *PLoS ONE*, namely *Scientific Reports*, marked the strongest asymmetry against *Psychological Research* (less often being cited than citing *Psychological Research*), followed by *Human Movement Science* and *Journal of Motor Behavior*. Close to that extreme was also a high-impact neuroscience journal, namely *Trends in Neuroscience*. In short, I was not able to identify any conspicuous feature of journals that at least in part could account for the observed citation asymmetries.

## From the past into the future

*Psychological Research/Psychologische Forschung* has survived a century of crises of various kinds. Notorious are the crises of Psychology, which have even found an entry in the current 19th edition of the traditional German “Psychologisches Wörterbuch”, the “Dorsch”. This dictionary, which is now called “Lexikon der Psychologie”, started with the first edition in 1921 and thus shares the century with the Journal. According to Fahrenberg (2020), the author of the entry “Krise der Psychologie”, publications on psychology in crisis started already in 1899, with the most popular treatment being that by Bühler (1927). In Germany there was an obvious crisis of academic psychology during the Nazi regime and thereafter, which is commonly attributed to the forced emigration of leading scientists. However, there has also been the thesis that major schools of Psychology such as the Berlin and the Leipzig school had passed their climax already before that time (Prinz, 1985; cf. Scheerer, 1988). In the second halve of the twentieth century, Experimental Psychology only slowly recovered in Germany, as did the Journal. *Psychologische Forschung/Psychological Research* has never been strongly influenced by behaviorism. Thus, for the Journal the so-called cognitive revolution was more a smooth continuation than a revolution. As it appears to me, the Journal has published a steady flow of scientific advancements, and in the post-war period, at least after a certain level of recovery, it has not been shaken by crises or paradigm shifts. As was the case from the very start, the Journal—as stated in the “Aims and Scope”—is still “devoted to the dissemination of knowledge based on firm experimental ground, but not to particular approaches or schools of thought”.

The current crisis of psychology—among other sciences—is the replication crisis which might shake the “firm experimental ground” on which the Journal is built. As it turns out, the experimental ground in general is not really firm but somewhat muddy. The main reason is the uncertainty of statistical inferences—there is a probability above zero that they are wrong, and with an increasing number of researchers and studies the number of wrong inferences does necessarily increase. The problem with wrong inferences is aggravated by the available computational power that allows trying different analyses quickly to identify a reportable one. This is not the place to enter into a discussion of the replication crisis, but it should not cause major problems for *Psychological Research*. The reason is that the crisis seems to apply primarily to “groundbreaking” research and less so to research of a more “incremental” nature. These are buzz words, and at least the word “incremental” is usually used pejoratively in contrast to “novel” (cf. Marder, 2020) or even “groundbreaking”. Nevertheless, in my mind *Psychological Research* is a journal for incremental research—perhaps better called “cumulative research” to avoid the pejorative connotations—along a straight road that leads to the horizon and beyond. Thus, new findings generally should be in line with previous findings and/or plausible theories. Conspicuous deviations from the straight road, findings that are unexpected from the perspective of previous findings and theorizing, that are at variance with what is reasonable for the common sense or an ‘experimentally trained mind’, invite the suspicion of future replication problems.

The buzz words discussed by Marder (2020) illustrate a jargon that is currently in use by reviewers of manuscripts (and research proposals etc.), and they are reminding of a number of changes in the submission and review procedures during the last few decades. At least some of them seem to be related to the replication crisis and to increasing concerns about scientific misconduct. Thirty or 40 years ago I submitted a paper by mail, accompanied by a letter saying that I would appreciate if it could be considered for publication in < name of a journal > . A few weeks later a letter with reviews and revision requests arrived, or the manuscript was returned and rejected. By now there are a few obvious changes. Typically submission is via an editorial system that requests various kinds of information and declarations. (I have to admit that I have successfully avoided these systems for several years by now—thanks to caring co-authors.) Some of the requests, such as the declaration that the manuscript is not submitted elsewhere or that the data are accessible somehow, appear to me as marks of mistrust. Others, such as the information on the individual contributions of the authors, may not always be answered truthfully, and categories such as “conceived and designed the experiments” can cover quite diverse sorts of contributions. Information about statistical power, usually a justification for the sample size of the study, often appears somewhat arbitrary. Finally, being asked for the suggestion of reviewers or for a letter that praises the virtues of the manuscript may make the editor’s job easier, but does not support an as objective as possible judgment of the quality of the manuscript. For the editors it has become harder to find reviewers as the declines of review requests seem to increase in frequency. This might be related to the increasing frequency of more than one round of reviewing and revision, which sometimes can go on until all reviewers are satisfied (or fed up with the manuscript).

The changes of submission and review procedures certainly have to do with the increasing load placed on editors by an increasing number of submissions, with the increasing number of review requests, and with attempts to keep instances of non-replicable findings and scientific misconduct away from a journal. Somehow the system differs from what appears to me as a sort of ideal: editors who are knowledgeable in the field for which they handle manuscripts, know which reviewers to invite and how to weigh their comments and to evaluate the revision of the manuscript; reviewers who accept review invitations and can expect to write one review in due time, and not two or more reviews of successive revisions. This clearly is not the world we live in, but wanting editorial systems to run like this may not just be an old man’s longing for the past, but a desideratum for the future.
